# Association of Systemic Inflammation with Inflammatory mRNA Expression in Visceral Adipose Tissue in Gestational Diabetes

**DOI:** 10.3390/metabo15100644

**Published:** 2025-09-26

**Authors:** Renata Saucedo, María Isabel Peña-Cano, Mary Flor Díaz-Velázquez, Alejandra Contreras-Ramos, Miranda Moleres-Orduña, Debbie López-Sánchez, Jorge Valencia-Ortega, Javier Pérez-Duran

**Affiliations:** 1 Unidad de Investigación Médica en Enfermedades Endocrinas, Hospital de Especialidades, Centro Médico Nacional Siglo XXI, Instituto Mexicano del Seguro Social, Mexico City 06720, Mexico; renata.saucedo@imss.gob.mx; 2 Hospital de Ginecología y Obstetricia 221, Instituto Mexicano del Seguro Social, Toluca 50150, Mexico; isabelpenacano@hotmail.com; 3 Hospital de Ginecología y Obstetricia 3, Centro Médico Nacional La Raza, Instituto Mexicano del Seguro Social, Mexico City 02990, Mexico; mary.diaz@imss.gob.mx; 4 Laboratorio de Investigación en Biología Molecular, Hospital Infantil de México “Federico Gómez”, Mexico City 06720, Mexico; acora_ramos@hotmail.com; 5 Terapia de Mínima Invasión y Alojamiento Conjunto, Instituto Nacional de Perinatología “Isidro Espinosa de los Reyes”, Mexico City 11000, Mexico; mirandamoleres@gmail.com; 6 Sección de Estudios de Posgrado, Escuela Superior de Medicina, Instituto Politécnico Nacional, Mexico City 11340, Mexico; debbiearleth21@gmail.com; 7 Departamento de Patología, Centro Médico Naval, Mexico City 04830, Mexico; 8 Departamento de Investigación en Salud Reproductiva y Perinatal, Instituto Nacional de Perinatología “Isidro Espinosa de los Reyes”, Mexico City 11000, Mexico

**Keywords:** gestational diabetes, inflammation, visceral adipose tissue

## Abstract

**Background/Objectives**: Gestational diabetes mellitus (GDM) is characterized by a systemic inflammatory response and the expression of inflammatory factors in visceral adipose tissue (VAT). However, the association between these two inflammatory processes has not been fully elucidated. Therefore, this study aimed to (1) investigate whether whole blood counts, the neutrophil–lymphocyte ratio (NLR), the monocyte–lymphocyte ratio (MLR), serum adiponectin levels, and the mRNA expression of inflammatory genes (TLR2, TLR4, pro-inflammatory cytokines: IL-1β, IL-6, and TNF-α, anti-inflammatory cytokines: IL-1RA, IL-10, and adiponectin) in VAT are altered in women with GDM in comparison to pregnant women with normal glucose tolerance (NGT), and (2) determine the correlations between systemic and local VAT inflammation in all, GDM, and NGT women. **Methods**: Study of 50 GDM and 50 women with NGT with a cross-sectional design. Standard biochemical and hematological tests were conducted and relative mRNA expression in VAT was measured by RT-qPCR. **Results**: Women with GDM showed higher neutrophil, monocyte, NLR, MLR, and VAT TNF-α/IL-10 mRNA expression ratios while lymphocyte and eosinophil counts, serum adiponectin, and mRNA local VAT inflammatory markers such as TLR2, TLR4, IL-1β, IL-6, IL-1RA, and IL-10 were lower in women with GDM relative to women with NGT. Additionally, the circulating monocyte count were associated with TLR2 and TLR-4 VAT mRNA expression levels and eosinophils count were associated with IL-1β, IL-6, IL-10, and IL-1RA VAT expression levels in women with GDM. **Conclusions**: GDM is characterized by systemic inflammation, and some circulating immune cells, such as monocytes and eosinophils, are associated with the expression of inflammatory markers in VAT.

## 1. Introduction

According to the criteria established by the International Association of Diabetes in Pregnancy Study Group (IADPSG), gestational diabetes mellitus (GDM) is the primary endocrine disorder that occurs during pregnancy, affecting 14% of all pregnant individuals globally [1]. The prevalence of this condition has increased over the past decades [2]. GDM is associated with adverse short- and long-term outcomes for both mothers and their offspring. Women are at a higher risk of developing hypertensive disorders and requiring a C-section. Adverse neonatal outcomes include macrosomia, hypoglycemia, shoulder dystocia, preterm delivery, and respiratory distress [3,4]. Long-term sequelae for the mother and offspring include type 2 diabetes mellitus (T2DM) and cardiovascular disease [5].

Notably, GDM is an inflammatory condition. Circulating inflammatory cells and pro-inflammatory cytokines are up-regulated in GDM and are involved in the development of insulin resistance and pancreatic β-cell failure [6]. Cytokines are primarily produced by immune system cells; however, the placenta and adipose tissue are also sources of them [7]. In addition to whole blood counts and cytokines, systemic inflammatory indices such as the neutrophil–lymphocyte ratio (NLR) and monocyte–lymphocyte ratio (MLR) are associated with GDM [8].

Obesity is a major risk factor for the development of GDM, and it exacerbates the inflammatory state of pregnancy [9,10]. A cross-link has been identified between systemic inflammation and the release of mediators from adipose tissue. It has been reported that circulatory immune cells such as monocytes, lymphocytes, and neutrophils infiltrate adipose tissue, contributing to adipose tissue inflammation [11]. On the other hand, adipokines, which are secreted by macrophages and adipocytes, act in an autocrine fashion, further exacerbating systemic and adipose tissue inflammation [12]. These changes are accompanied by the down-regulation of adipokines such as adiponectin, which has an anti-inflammatory action [13].

Visceral adipose tissue (VAT) exhibits a more pro-inflammatory expression profile during pregnancy and contains a higher number of lymphocytes in comparison to subcutaneous fat [14]. Women with GDM have been shown to have increased visceral adiposity [15]. A recent systematic review and meta-analysis revealed a positive correlation between visceral adiposity and the risk of developing GDM [16]. It has been suggested that VAT might play a role in the etiopathogenesis of GDM through expression of genes associated with insulin resistance, lipid metabolism, and inflammation [17,18]. However, to date, only a limited number of studies have concurrently described the systemic and local inflammatory status in VAT of women with GDM. Therefore, we sought to (1) investigate whether whole blood counts, systemic inflammatory indices (NLR and MLR), serum adiponectin levels, and inflammatory gene expression in VAT (pattern recognition toll-like receptors: TLR2 and TLR4; pro-inflammatory cytokines: IL-1β, IL-6, and TNF-α; anti-inflammatory cytokines: IL-1RA, IL-10, and adiponectin) are altered in GDM compared to in pregnant women with normal glucose tolerance (NGT), and (2) determine correlations between systemic and local VAT inflammation in all, GDM, and NGT women. We hypothesized that participants with GDM would have systemic inflammation and that this would be related to the local VAT inflammatory state.

## 2. Materials and Methods

### 2.1. Study Population

The study was conducted in accordance with the principles of the Declaration of Helsinki and was approved by the Institutional Review Board of the Instituto Mexicano del Seguro Social (IMSS) in Mexico City (R-2018-785-026). All the participants provided written informed consent. 

The present study employs a comparative cross-sectional design. The study population comprised 50 pregnant women diagnosed with GDM and an equal number of women with NGT, matched for age. Eligible participants were women aged at least 18 years with a singleton pregnancy who were scheduled for an elective cesarean section at term (>37 weeks’ gestation). The IADPSG criteria were used to define GDM based on a 2 h, 75 g oral glucose tolerance test between 24 and 28 weeks of gestation. Women were diagnosed with GDM if one or more of the following plasma glucose values were met or exceeded [fasting plasma glucose (FPG) of 92 mg/dL (5.11 mmol/L) and/or 1 h and/or 2 h OGTT values of 180 mg/dL (10.00 mmol/L) or 153 mg/dL (8.5 mmol/L), respectively] [1]. In the GDM group, 36% (*n* = 18) were treated only with diet, 22% (*n* = 11) with diet plus insulin, 34% (*n* = 17) with diet plus metformin, and 8% (*n* = 4) with a combination of diet, insulin, and metformin. Exclusion criteria for all women included pregnancies resulting from assisted reproductive technology, chronic diseases, infections, multiple pregnancies, and alcohol abuse. The demographic and maternal history information of each participant was obtained from medical records and a questionnaire.

### 2.2. Analytical Methods

Before the scheduled cesarean section and after an overnight fast, a maternal blood sample was collected in a 5 mL EDTA tube and in a 10 mL red blood serum tube. The samples in red tubes were allowed to clot for at least 30 min before 15 min of centrifugation at 1000× *g*, and serum was obtained for determination of biochemical assays or stored at −80 °C until adiponectin and insulin analysis. Whole blood counts were determined in an EDTA tube using the automated blood cell counter Cell-Dyn Ruby (Abbott Laboratories, Abbott Park, IL, USA). NLR and MLR were calculated by dividing absolute blood neutrophil counts and monocyte counts by absolute lymphocyte counts, respectively.

Determination of biochemical assays was carried out using an ARCHITECT Plus c4000 Clinical Chemistry Analyzer (Abbot Laboratories, Abbott Park, IL, USA). Levels of low-density lipoprotein cholesterol (LDL) were calculated using the Friedewald formula. Serum adiponectin and insulin were measured by multiplex immunoassay using Magpix technology (Milliplex Map, Billerica, MA, USA) following the manufacturer’s instructions. The inter- and intra-assay coefficients of variation were all found to be less than 10%. The homeostasis model assessment of insulin resistance (HOMA-IR) index = fasting glucose (mmol/L) × fasting insulin (mU/L)/22.5 was used in the present study.

### 2.3. VAT mRNA Extraction and cDNA Synthesis

Ten minutes after delivery, VAT was collected, washed in water treated with diethylpyrocarbonate to remove blood, and dissected into fragments. The fragments were placed in TRIzol^®^ Reagent (Invitrogen™, Carlsbad, CA, USA) and stored at −70 °C until RNA extraction. RNA was extracted using Direct-zol™ RNA MiniPrep kit (Zymo Research Corp, Tustin, CA, USA), according to the manufacturer’s instructions. RNA was quantified using the NanoDrop 2000 spectrophotometer (Thermo Fisher Scientific, Newark, DE, USA), and the A260/A280 ratio was used to determine RNA quality. A total of 1 µg RNA was reverse-transcribed to cDNA using the SuperScript^®^III FirstStrand kit (Invitrogen™, Carlsbad, CA, USA), following the manufacturer’s instructions.

### 2.4. Quantitative Real-Time RT-PCR

Inflammatory mRNA expression was quantified using real-time PCR with TaqMan^®^ Gene Expression Assays and TaqMan^®^ Universal PCR Master Mix (Applied Biosystems™, Foster City, CA, USA), as described previously [19]. Real-time PCR was performed with the StepOnePlus™ Real-Time PCR System (Applied Biosystems™, Foster City, CA, USA). The 2^−ΔCt^ method of relative quantification was used to determine the fold change in the mRNA expression with the GAPDH transcript as endogenous control. All the primers and probes were acquired from Applied Biosystems™: TLR2 (Hs02621280_s1), TLR4 (Hs00152939_m1), IL-1β (Hs01555410_m1), IL-6 (Hs00985639_m1), TNF-α (Hs01113624_g1), IL-1RA (Hs00893626_m1), IL-10 (Hs00961622_m1), adiponectin (Hs00977214_m1), and GAPDH (PN 4326317E).

### 2.5. Statistical Analysis

Sample size calculations were performed using G*Power 3.1.9.6 software from Faul et al. [20], based on previous literature comparing whole blood counts between women with GDM and women with NGT. A sample size of 50 participants per group was calculated, with an alpha level of 0.05 and a power of 0.80. Median (P25, P75) was used to represent continuous variables. These variables were compared between the groups using the Mann–Whitney U test. Associations between measures were examined using Spearman correlation coefficients. The correlation between inflammatory cells with GDM was analyzed by logistic regression analyses adjusting for age, pregravid body mass index (BMI), and week of gestation. Finally, to analyze the independence of the association between mRNA expression and systemic inflammation, a multiple linear regression model was developed; age, pregravid BMI, week of delivery, and systemic inflammation were included as independent variables, and mRNA expression levels were included as the dependent variable. Data analysis was conducted with IBM SPSS version 23.0 (IBM Co., Armonk, NY, USA) software. A value of *p* < 0.05 was considered significant.

## 3. Results

### 3.1. Characteristics of the Participants

As shown in Table 1, women with GDM had higher BMI before pregnancy, glucose, neutrophil count, monocyte count, NLR, and MLR compared with women with NGT. In addition, patients with GDM had lower gestational age, total cholesterol, high-density lipoprotein cholesterol (HDL), lymphocyte count, eosinophil count, and serum adiponectin levels than women without GDM. The differences that continued to be significant after adjusting for pre-pregnancy BMI, BMI at term, gestational age at delivery, and age were monocytes, eosinophils, NLR, MLR, and HDL. There was no difference in age, maternal weight gain, triglycerides, LDL, very low-density lipoprotein (VLDL), insulin, HOMA-IR, leukocyte, and basophil count between women with GDM and those with NGT. In adjusted multivariable regression, NLR and MLR were significantly associated with GDM (OR 1.71 95% CI 1.03–2.83; OR 1.4 95% CI 1.17–3.6, respectively). In addition, there was no significant difference in whole blood counts, NLR, MLR, and serum adiponectin levels between women with GDM who were managed with dietary modification alone and those who were treated with insulin or metformin.

### 3.2. Gene Expression in VAT from the Patients with and Without GDM

mRNA inflammatory VAT expression levels are shown in Table 2. The GDM group showed lower mRNA TLR2, TLR4, IL-1β, IL-6, IL-1RA and IL-10 than NGT. The only significance that remained after adjusting was IL-10. No difference was observed between the groups with respect to TNF-α and adiponectin mRNA expression in VAT. To investigate the pro-inflammatory cytokine/anti-inflammatory cytokine balance, we observed that VAT TNF-α/IL-10 expression ratio was higher in GDM compared with NGT (*p* = 0.002). Moreover, there was no significant difference in cytokine expression between women with GDM which was managed with dietary modification alone and those who received pharmacotherapy.

### 3.3. Correlations Between Inflammation VAT Gene Expression as Well as Maternal Clinical Parameters and Systemic Inflammation

Table 3 summarizes significant correlations between the studied variables in all patients. There was a significant correlation for eosinophil count with TLR2, IL-1β, IL-6, TNF-α, IL-1RA, and IL-10, lymphocyte count with IL-1β and IL-1RA, neutrophil count with IL-6, and NLR with TLR2, IL-6, and IL-1RA.

In women with GDM, there was a correlation for monocytes with TLR2 (r = 0.343, *p* = 0.024) and TLR4 (r = 0.347, *p* = 0.022), and for eosinophils with IL-1β (r = 0.416, *p* = 0.006), IL-6 (r = 0.314, *p* = 0.040), IL-10 (r = 0.417, *p* = 0.005), and IL-1RA (r = 0.315, *p* = 0.039). In adjusted multiple linear regression analysis, TLR2 remained significantly associated with monocytes (r^2^ = 0.382, β = 0.333, *p* = 0.026). In NGT, there were no associations. 

Additionally, serum adiponectin levels were associated with BMI at delivery (r = −0.419, *p* = 0.05) and with NLR (r = −0.518, *p* = 0.023) and MLR (r = −0.688, *p* = 0.001) in all the participants. However, after adjusting for BMI, age, and weeks of gestation, these associations disappeared.

## 4. Discussion

In this study, inflammatory blood cell parameters, such as neutrophil count, monocyte count, NLR and MLR, and VAT TNF-α/IL-10 expression ratio, were higher in at-term women with GDM relative to age-matched women with NGT. In contrast, lymphocyte and eosinophil count, serum adiponectin, and mRNA local VAT inflammatory markers such as TLR2, TLR4, IL-1β, IL-6, IL-1RA, and IL-10 were lower in women with GDM than without. Additionally, circulating monocyte counts were associated with TLR2, and TLR4 VAT expression levels and eosinophil counts were associated with IL-1β, IL-6, IL-10, and IL-1RA VAT expression levels in women with GDM. Consistent with the first observation, previous studies have reported that neutrophils are elevated in GDM cases compared to NGT, and that their count during early pregnancy constitutes an independent risk factor and predictive factor for GDM [21,22]. It has been suggested that neutrophils play a role in the development of GDM by contributing to insulin resistance through the secretion of elastase [23]. On the other hand, we showed that monocyte count is significantly increased in women with GDM than in women with NGT, a difference that remains significant after adjusting for confounding factors. Our results are in line with earlier research, which has shown that human peripheral monocytes are linked to GDM, but ran counter to others reporting decreased monocyte count in GDM [24,25]. Sahin et al. found elevated monocyte counts in GDM [22]. On the contrary, Liu et al. indicated that monocyte blood levels were significantly lower in a GDM group than in a NGT group [26]. The authors further reported that, among all the inflammatory factors they investigated, monocytes had the highest diagnostic level for GDM, with an area under the curve of 0.619, and a sensitivity and specificity of 70.61% and 50.15%, respectively [26]. The discrepancies in the results may be related to the measurement methods and the participant selection criteria.

The relationship between lymphocytes and GDM is controversial. In our study, women with GDM showed lower lymphocyte count compared with the NGT group. Some studies are in agreement with our results; however, several studies have indicated a higher lymphocyte count in GDM compared to NGT [22,27]. The discrepancies could be related to the gestational age of the participants. Simsek et al. showed a higher lymphocyte count during the first trimester than before delivery in women with GDM, and similarly, we evaluated lymphocyte count during late gestation [28].

According to the current study, eosinophil count was significantly lower in the GDM group compared to pregnant women with NGT, a difference that persisted after controlling for covariates. No reason was encountered in the literature on this subject. However, the inflammatory state in GDM might be the reason. Inflammatory cells induce the migration of eosinophils from the blood into the affected tissues [29]. In this respect, a recent bioinformatics analysis of GDM placentas confirmed that, among 28 immune cell types, only eosinophil infiltration scores differed significantly between GDM and NGT [30]. Additionally, an overexpression of galectin-10, a cytoplasmic protein of human eosinophils, has been identified in GDM placentas [31]. Furthermore, there are similar findings in another inflammatory gestational complication, preeclampsia; Gelaw et al. [32] revealed that eosinophil count was significantly lower in women with preeclampsia compared to normotensive pregnant women.

NLR and MLR are two other inflammatory markers that can easily be calculated. Several studies have suggested that an elevated NLR might be an effective marker to predict GDM and its complications [33]. Wang et al. found elevated NLR in patients with GDM at diagnosis [33]. Liu et al. showed that increased NLR was an independent predictor of GDM [34]. Hessami et al. and Pace et al. found in their meta-analysis that NLR was associated with GDM [35,36]. Furthermore, the prognostic value of increased NLR in T2DM and cardiovascular diseases has been well described in the literature [37,38]. In addition, it has been reported that MLR is a good predictor of GDM, and this index also has diagnostic value in predicting maternal–fetal complications [33]. Wang et al. concluded that elevated MLR is associated with the development of GDM [33]. Baki et al. found that a higher MLR was independently associated with the risk of GDM [39]. Liu et al. demonstrated that MLR was significantly correlated with the development of GDM [26]. In our study, NLR and MLR were higher in women with GDM compared to women with NGT, and both were predictors of GDM.

Adiponectin is an adipokine that stimulates insulin secretion and has insulin-sensitizing properties. This study found lower serum adiponectin levels in GDM compared to pregnant women with NGT, which was consistent with previous studies but contrary to others that reported unchanged adiponectin levels in women with GDM [40,41]. The observed discrepancies in the results may be attributable to the utilization of diagnostic criteria to define GDM, gestational age at blood sampling, differences in ethnicity and BMI of the women, and assay methods for adiponectin measurement. We also showed that the difference did not remain significant after adjustment for age and BMI. Furthermore, circulating adiponectin correlated negatively with BMI at delivery and was not associated with insulin and HOMA-IR, suggesting that adiponectin levels appear to be more related to maternal BMI than to the insulin-resistant state. In addition to its metabolic functions, adiponectin also exerts anti-inflammatory properties; it inhibits TNF-α in macrophages, induces the expression of IL-10, and regulates innate immunity, suppressing the activation of eosinophils, neutrophils, and lymphocytes in vitro [12,13]. Furthermore, adiponectin has been demonstrated to induce a shift in macrophages from the M1 to M2 phenotype, thereby reducing chronic inflammation [42]. Interestingly, the present study found an inverse relationship between serum adiponectin levels and NLR and MLR in all subjects. However, these associations were lost after adjusting for confounding factors, including BMI. These results suggest that adiponectin levels are related to adipose tissue mass rather than to inflammatory dysregulation in GDM. Similarly, the only other study of GDM reported no association between NLR and adiponectin levels at either the time of diagnosis or birth in patients with GDM [43].

The current study also determined inflammatory gene expression in VAT of GDM compared to women with NGT. The results showed that the GDM group had lower mRNA TLR2, TLR4, IL-1β, IL-6, IL-1RA, and IL-10 than the NGT group. However, VAT TNF-α/IL-10 expression ratio was higher in GDM compared to NGT. The only significant difference that remained after adjusting was IL-10. These findings could reflect the immune imbalance in GDM characterized by an altered anti-inflammatory status. This is in agreement with Kuzmicki et al. [44], who demonstrated a down-regulation of serum IL-10 in GDM. On the other hand, we did not observe a difference between GDM and NGT with respect to adiponectin mRNA expression in VAT, and there was no association of serum adiponectin with its mRNA expression in VAT. The above findings agree with those of Kleibova et al. [45], who reported that expression of adiponectin mRNA did not significantly differ in VAT of patients with GDM compared with NGT. However, our results were contrary to other studies reporting decreased expression of adiponectin in VAT in GDM compared with non-GDM pregnancies [18,46,47,48]. The inconsistencies in the results might be because of ethnic origin, BMI, and treatment of GDM in the subjects.

Mothers with GDM have a more inflammatory profile in VAT than those with NGT [18]. It has been suggested that adipose tissue inflammation is sustained over time by infiltration of bone marrow-derived immune cells [49]. Some studies have shown differences in lymphocyte phenotype and more macrophages in VAT of GDM compared to normal pregnancies [14,50]. The infiltration of inflammatory cells correlates with increased expression of inflammatory mediators and with metabolic dysfunction. In this study, positive correlations between monocyte count and TLR2 and TLR4 mRNA expression levels were observed in GDM. TLRs are expressed in numerous types of cells, including adipose cells and monocytes, and are initiators of innate immunity, recognizing preserved patterns as pathogen-associated lipopolysaccharides and other lipid moieties, and then triggering cellular signals, culminating in the activation of NF-κB and in the induction of inflammatory responses. In the current study, adjusted multiple linear regression analysis showed that TLR2 remained significantly associated with monocytes in GDM. This finding may reflect a higher monocyte infiltration in the VAT of women with GDM, which can mature into macrophages. Interestingly, Dong et al. described a greater macrophage infiltration in the VAT of pregnant women with GDM than in women without GDM [46]. Additionally, a recent publication reported an increase in a classical tissue monocyte cluster in the VAT of obese participants with GDM [51].

We also showed a relationship between eosinophil count and IL-1β, IL-6, IL-10 and IL-1RA expression levels in the GDM group. This is consistent with eosinophils’ role in regulating immune functions. It has been reported that eosinophils rapidly leave the bloodstream to enter tissues as adipose tissue, where they produce and secrete over 30 cytokines [52].

The present study has some limitations. The sample size was modest, and the groups were not matched with respect to BMI. All GDM patients received an intervention upon diagnosis, which included dietary management, insulin, and metformin treatments, so the findings might not apply to other populations because pharmacological intervention may have impacted adipocyte function and inflammation. Also, the VAT analyses were on whole tissue samples instead of isolated adipocytes or leukocyte populations. Furthermore, we did not measure VAT immune cell population content. Finally, the cross-sectional design of this study cannot provide causality. Therefore, it would be appropriate to evaluate, in cohort studies with a larger sample size, the role of systemic inflammation in VAT inflammatory patterns of GDM subjects.

## 5. Conclusions

We provide evidence that systemic inflammation, which we investigated by circulating neutrophil and monocyte count, and NLR and MLR, is increased in at-term women with GDM. Additionally, we observed a higher VAT TNF-α/IL-10 mRNA expression ratio and lower mRNA expression of TLR2, TLR4, IL-1β, IL-6, IL-1RA, and IL-10 in women with GDM relative to women with NGT. These findings, together with the association of circulating monocyte count with TLR2 and TLR-4 VAT mRNA expression levels and eosinophil count with IL-1β, IL-6, IL-10, and IL-1RA VAT expression levels in women with GDM, suggest that systemic inflammation is accompanied by dysregulated VAT expression of inflammatory genes in women with GDM.

## Figures and Tables

**Table 1:** Clinical and biochemical characteristics of the study groups.

	NGT (*n* = 50)	GDM (*n*= 50)	*p*
Age (year)	30.0 (22–33)	29.0 (27–33)	0.353
BMI before pregnancy (kg/m^2^)	25.6 (23.2–27.3)	29.5 (26.7–33.9)	0.001
GWG (kg)	10 (7.8–11.6)	9 (6.8–11.0)	0.332
Gestational age (weeks)	39.0 (38.0–39.6)	38.0 (37.5–39.0)	0.013
Fasting glucose (mmol/L)	4.3 (3.8–5.1)	4.8 (4.1–5.5)	0.040
Total cholesterol (mmol/L)	6.1 (5.2–8.3)	5.4 (4.8–6.5)	0.036
Triglycerides (mmol/L)	3.1 (2.5–3.6)	3.1 (2.2–3.8)	0.691
HDL (mmol/L)	2.7 (2.3–3.1)	2.4 (1.9–2.8)	0.039
LDL (mmol/L)	1.9 (1.2–2.3)	1.4 (0.8–2.2)	0.122
VLDL (mmol/L)	1.4 (1.2–1.7)	1.4 (1.0–1.8)	0.537
Insulin (pmol/L)	51.8 (30.0–73.4)	53.8 (34.4–79.3)	0.827
HOMA-IR	1.3 (0.82–2.1)	1.6 (1.1–2.5)	0.224
Leukocyte count (×10^9^/L)	8.8 (7.7–9.7)	8.9 (8.2–9.9)	0.192
Neutrophil count (×10^9^/L)	5.7 (5.1–6.7)	6.5 (5.4–7.5)	0.035
Lymphocyte count (×10^9^/L)	2.3 (1.9–2.7)	2.0 (1.7–2.4)	0.021
Monocyte count (×10^9^/L)	0.5 (0.4–0.6)	0.6 (0.5–0.7)	0.027
Eeosinophil count (×10^9^/L)	0.10 (0.08–0.14)	0.06 (0.03–0.10)	0.001
Basophil count (×10^9^/L)	0.03 (0.02–0.04)	0.02 (0.02–0.03)	0.441
NLR	2.5 (2.1–2.9)	3.2 (2.5–4.2)	0.001
MLR	0.22 (0.16–0.27)	0.29 (0.21–0.37)	0.001
Adiponectin (pg/mL)	253.3 (68.3–342.4)	69.0 (29.2–169.6)	0.002

Data are presented as median (Q1–Q3). NGT: normal glucose tolerance; GDM: gestational diabetes mellitus; BMI: body mass index; GWG: gestational weight gain; HDL: high-density lipoprotein cholesterol; LDL: low-density lipoprotein cholesterol; VLDL: very low-density lipoprotein; HOMA-IR: homeostasis model assessment of insulin resistance; NLR: neutrophil–lymphocyte ratio; MLR: monocyte–lymphocyte ratio.

**Table 2:** Inflammatory marker mRNA expression (2^−^^ΔCt^) in VAT of the NGT and GDM groups.

	NGT (*n* = 50)	GDM (*n*= 50)	*p*
TLR2	0.040 (0.014–0.094)	0.015 (0.008–0.038)	0.002
TLR4	0.067 (0.040–0.096)	0.033 (0.011–0.104)	0.017
IL-1β	0.415 (0.220–0.859)	0.231 (0.106–0.541)	0.012
IL-6	0.354 (0.146–0.915)	0.126 (0.043–0.412)	0.002
TNF-α	0.022 (0.012–0.031)	0.022 (0.009–0.033)	0.767
IL-1RA	0.042 (0.023–0.088)	0.028 (0.014–0.068)	0.052
IL-10	0.104 (0.060–0.218)	0.048 (0.023–0.131)	0.001
Adiponectin	0.286 (0.121–1.032)	0.276 (0.116–1.200)	0.825

Data are presented as median (Q1–Q3). VAT: visceral adipose tissue; NGT: normal glucose tolerance; GDM: gestational diabetes mellitus; TLR: toll-like receptor; IL: interleukin; TNF-α: Tumor necrosis factor-alpha.

**Table 3:** Significant correlations between studied variables in all women.

	r	*p*
	Eosinophil count	
TLR2	0.263	0.012
IL-1β	0.368	0.001
IL-6	0.280	0.007
TNF-α	0.214	0.042
IL-1RA	0.305	0.003
IL-10	0.344	0.001
	Lymphocyte count	
IL-1β	0.215	0.041
IL-1RA	0.231	0.027
	Neutrophil count	
IL-6	−0.211	0.043
	NLR	
TLR2	−0.259	0.013
IL-6	−0.257	0.013
IL-1RA	−0.274	0.009

TLR: toll-like receptor; IL: interleukin. TNF-α: Tumor necrosis factor-alpha. NLR: neutrophil–lymphocyte ratio.

## Data Availability

The original contributions presented in this study are included in the article. Further inquiries can be directed to the corresponding author(s).
